# Treatment of Hemorrhoids by Injections

**Published:** 1885-10

**Authors:** 


### The Treatment of Hemorrhoids by Injection.

In an instructive clinical paper in the July number of *The American Journal of the Medical Sciences*, Dr. Charles B. Kelsey, of New York, urges the treatment of hemorrhoids by injection of carbolic acid. After an ample experience, this has become his routine practice, and in all his cases he has never known a patient to abandon the treatment after it was begun, and he has never failed to effect a perfectly satisfactory cure by it, and he has never had an accident of serious nature with it. He uses three solutions, one of 15 per cent., one of 33 per cent., one of 50 per cent., and sometimes he uses the pure acid. In a severe case, he begins with the stronger ones; in a mild case, with the weaker.

---

A writer on medical education, in the last number of the *Popular Science Monthly*, commenting on the popular interest in medical topics, fostered by medical bulletins from the sick-beds of great men, and especially on the fascination which the germ theory seems to have for everybody at the present time, remarks that in the wilds of the West a cow-boy recently shot another for calling him a d—d microbe.—*Boston Medical and Surgical Journal.*

